# Establishment of injury models in studies of biological effects induced by microwave radiation

**DOI:** 10.1186/s40779-021-00303-w

**Published:** 2021-02-18

**Authors:** Yun-Fei Lai, Hao-Yu Wang, Rui-Yun Peng

**Affiliations:** Beijing Institute of Radiation Medicine, Beijing, 100850 China

**Keywords:** Microwave radiation, Injury model, Biological effects, Methods, Biological indicators, Review

## Abstract

Microwave radiation has been widely used in various fields, such as communication, industry, medical treatment, and military applications. Microwave radiation may cause injuries to both the structures and functions of various organs, such as the brain, heart, reproductive organs, and endocrine organs, which endanger human health. Therefore, it is both theoretically and clinically important to conduct studies on the biological effects induced by microwave radiation. The successful establishment of injury models is of great importance to the reliability and reproducibility of these studies. In this article, we review the microwave exposure conditions, subjects used to establish injury models, the methods used for the assessment of the injuries, and the indicators implemented to evaluate the success of injury model establishment in studies on biological effects induced by microwave radiation.

## Background

The World Health Organization (WHO) has listed electromagnetic radiation as one of the most common and fastest growing environmental influences [1]. Microwave radiation is a form of electromagnetic waves, with frequencies ranging from 300 MHz to 300 GHz. Microwave radiation has been widely used in various fields, such as communication, industry, medical treatment, and the military. Previous studies have shown that microwave radiation can cause injuries to both the structures and functions of the brain, heart, reproductive organs and endocrine organs, which endangers human health [2–9]. Studies on the biological effects induced by microwave radiation are essential for unveiling the mechanisms of these injuries and promoting the development of more efficient prevention methods and more profound treatment strategies. Successful establishment of injury models plays an important role in studies on the biological effects of microwave radiation. Not only is the successful establishment of injury models the premise of these studies, but it also has great importance to their reliability and reproducibility.

Generally, the establishment of injury models induced by microwave radiation requires stable microwave exposure conditions, suitable subjects, appropriate methods, and reliable biological indicators. The stable microwave exposure conditions promise the reproducibility of the microwave-radiation-induced biological effects [10]. Suitable subjects sensitive to specific microwave radiation injuries are essential in establishing different types of injury models. The appropriate methods are helpful in screening biological indicators sensitive to microwave radiation, which are important for assessing the successful establishment of injury models, understanding the underlying mechanisms of microwave radiation injuries, and laying foundations for corresponding clinical diagnosis and the development of targeted therapeutic drugs.

### Microwave exposure conditions

The microwave exposure conditions determine the reproducibility of the microwave-radiation-induced biological effects. The most commonly used parameters to depict the microwave exposure conditions in previous studies include the source frequency, the average incident power density, the specific absorption rate (SAR), the time variability and frequency variability, and the proximity of the subjects to the microwave exposure source.

The source frequency is one of the most important physical parameters of electromagnetic exposure. Microwave frequency bands, including the L band (1 - 2 GHz) [11], S band (2 - 4 GHz) [12–14], and X band (8 - 12 GHz) [15, 16], which are widely used in radar communication systems, were implemented in previous studies. In addition, frequencies such as 900 MHz [17–22], 1800 MHz [18, 22–24] (Global System for Mobile Communications signals), and 2450 MHz [2, 25–34] (microwave oven and WiFi equipment) were also implemented in related studies.

The average power density is another significant physical parameter of electromagnetic exposure. The average power densities of 2.5, 5, 10, 30, and 50 mW/cm^2^ were used to establish biological injury models to illuminate the relationship between the biological effects and the microwave radiation doses [4, 35–39]. For instance, Wang *et al*. [4] found that long-term microwave exposure (2.5 – 10.0 mW/cm^2^) could cause spatial learning and memory deficits in rats, which were positively correlated with the average power densities. In addition, a microwave exposure system with lower average power densities ranging from 10^-2^ mW/cm^2^ to 10^-1^ mW/cm^2^ was also used to establish biological injury models [40–42].

The SAR value is an internationally accepted electromagnetic radiation dosimetric parameter. The SAR value distribution in animals or cells relies not only on the frequency, incident direction, and E-polarization direction but also on the structure of the subject under exposure and the electromagnetic properties of different tissues. To date, the SAR values implemented in studies on microwave radiation biological effects range from 10^-4^ W/kg to 35 W/kg [12, 13, 18, 43].

The time variability on many time scales may influence the establishment of a microwave injury model. For example, microwave exposure can be classified into pulsed wave (PW) exposure and continuous wave (CW) exposure. Pulsed microwave exposure is characterized by its nonlinearity and instantaneous nature, which may be the reason why the adverse effects are more serious than those caused by continuous exposure [44]. Moreover, microwave exposure can also be classified as single exposure and multiple exposures based on the number of exposures. On the one hand, a single exposure was widely used in studies on biological effects after acute high-dose microwave radiation [38]. On the other hand, a multiple-exposure mode was implemented in studies on the biological effects of long-term low-dose microwave radiation [42, 45].

The frequency variability has also been studied. For instance, single-frequency and combined microwave exposures are classified according to their frequency domain properties. Most studies on microwave radiation exposure have focused on single-frequency exposure. In fact, simultaneous exposure to different frequency microwave radiation is closer to the real scenario. For example, an *in vivo* study reported that cognitive dysfunctions induced by combined exposure (1.5 GHz and 2.856 GHz) were more serious than those induced by single-frequency exposure [11].

The proximity of the subjects to the microwave exposure source is another parameter that may influence the establishment of a microwave radiation injury model, since the mechanism of subject heating is different in near-field and far-field exposures (mostly electrical field driven vs radiation absorption). In medical applications, near-field effects are predominant, whereas in telecommunications, far-field effects are predominant [46–53].

Other exposure conditions that could influence the establishment of a microwave radiation injury model, including but not limited to the modulation, the waveform, chemical cofactors, whole-animal or head-only exposure, the duration of exposure, and the time between exposure and measurement, have also been investigated [54–64].

### Subjects used to establish injury models

Appropriate subjects are the premises for establishing *in vivo* and *in vitro* injury models of microwave radiation. On the one hand, *in vivo* studies are crucial to explore the biological effects of microwave radiation in complex biological conditions. On the other hand, *in vitro* studies are often used to unveil the biological mechanisms. Since different subjects have different sensitivities to microwave radiation, the choice of animal species, cell types or other organisms should be determined by their special microwave sensitivities and the specific purpose of the study.

### Animal species

Various animal species (rats, mice, rabbits, monkeys, etc.) have been used in studies on the biological effects of microwave radiation. Each of these animal species has a unique advantage in studying the biological effects of microwave radiation on specific target organs or tissues.

#### Rats

Rats, including Wistar rats, Sprague-Dawley (SD) rats, and Fischer-344 rats, were widely used in studies on the biological effects of microwave radiation. Wistar rats were the most commonly used rodent for the detection of microwave-radiation-induced injury effects on target organs, such as the brain [3, 4, 12, 13, 17, 37, 65–78], heart [79–83], reproductive organs [79, 83–86], and endocrine organs [6]. SD rats were mainly used in studies on the effects of microwave radiation on the brain [87–103], heart [104–108], and skin [109]. In addition, Fischer-344 rats were used to evaluate the biological effects of microwave radiation on cognitive function [110–113] and the blood-brain barrier (BBB) [114–118].

#### Mice

Mouse species such as Kunming mice, Swiss mice, BALB/c mice, C57BL/6 mice, and NMRI mice were also used in studies of the effects of microwave radiation on brain function. Among these species, Kunming mice were mainly chosen by Chinese scholars in their studies [119, 120]. Swiss mice were more commonly used by scholars from other countries [2, 15, 16, 40, 95, 121–123]. In addition, BALB/c mice were also used to study the effects of microwave radiation on learning and memory functions. Furthermore, NMRI mice [124] and C57BL/6 mice [125, 126] were widely used to study the effects of microwave radiation on locomotor activity.

In addition, several studies have been conducted with transgenic heterozygous and heterozygous knockout mice, which are prone to specific tumors. These mice were used to study tumorigenesis induced by microwave radiation [127, 128].

#### Rabbits

It is well known that rabbits are sensitive to stress, especially during pregnancy. Pregnant female rabbits can miscarry easily when they are under stress [129–131]. Furthermore, rabbits easily yield semen at the appointed time, which is suitable for longitudinal studies [132]. Therefore, New Zealand white rabbits were used to investigate the biological effects of microwave radiation on pregnant women, the developmental stages of children’s brains from conception to childhood, and the male reproductive system [7, 129, 131–135].

Rabbits have also been used to establish microwave injury models of the brain, heart, spinal cord, and eye [136–139].

#### Monkeys

Since their corneas are similar to humans, rhesus monkeys (*Macaca mulatta*) were used to evaluate the bioeffects of microwave exposure on eyes [140–144]. The average thickness of the central cornea in rhesus monkeys is approximately 0.50 mm, which is close to that of humans (0.56 mm). Furthermore, similar to humans, in rhesus monkeys, the corneal endothelium is not capable of mitotic potential under pathological conditions [141].

#### Other animal species

Poultry eggs were implemented as typical animal models in biological effect studies of microwave radiation on embryonic development due to the well-developed nervous system and short feeding cycle of chickens [23, 145]. For instance, Yakymenko *et al*. [23] reported an increased embryo mortality of developing quail embryos after exposure to the Global System for Mobile Communication (GSM) signal of 1800 MHz.

Transgenic nematodes have also been used to establish microwave injury models [146].

### Cell types

Cell models are essential for studies on the biological mechanisms of microwave radiation injuries since they can exclude the influence of complex *in vivo* environments. The most widely used cell types in previous studies on microwave radiation injuries included neurons, germ cells, and heart cells.

#### Neurons

Neurons were often used in studies on brain injuries induced by microwave radiation. Previous studies implemented either primarily cultured neurons or neuron-like cell lines according to the specific scientific purposes and the advantages of different cell types. Primarily cultured neurons were isolated from *in vivo* rodent brains. Due to the indivisibility of mature neurons, they are not suitable for experiments that require a large number of cells. The primary cultured neurons used in previous studies on microwave radiation bioeffects included primary cortical neurons, hippocampal neurons, and astrocytes [147–150].

The neuron-like cell line mostly used in previous investigations of microwave radiation bioeffects was the PC12 cell line. The PC12 cell line is derived from rat adrenal pheochromocytoma cells with fast proliferation. They can be induced to generate a neuron-like cell type that has synapses when treated with nerve growth factor (NGF). The PC12 cell line is widely used to study the mechanisms of learning and memory after microwave exposure [36, 37, 67, 77, 151]. Moreover, HT22 cells [152, 153] and MN9D cells [154] were also used in studies on the biological effects of microwave radiation.

#### Germ cells

There are two main common germ cell types used in studies on the reproductive system of microwave radiation. One was the mouse spermatocyte line GC-2spd (ts) [155, 156], and the other was Sertoli cells [14]. The GC-2spd (ts) cell line derived from the cotransfection of mouse spermatocytes with the simian virus 40 (SV40) large T antigen gene and a temperature-sensitive mutant of the p53 gene is immortalized. The GC-2spd (ts) cell line expresses the lactate dehydrogenase C4 isozyme and the cytochrome c_t_ isoform. The GC-2spd (ts) line forms round spermatids [157]. Sertoli cells play a key role in the maintenance of normal spermatogenesis [158].

#### Heart cells

As the structural basis of cardiac excitatory contraction function, cardiomyocytes were often used to investigate the effects of microwave radiation on the myocardial cell membrane and intracellular calcium levels [80, 147, 159, 160]. Most studies showed that the intracellular calcium concentration decreased in primary cardiomyocytes exposed to microwave radiation at the frequencies of 2.856 and 9 GHz [147, 159], whereas the opposite finding was reported by Wolke *et al*. [160].

### Other organisms

Microwave radiation injury models are not restricted to the animal species and cell types mentioned above. Many different organisms, including even plants [161, 162], bacteria [163], and viruses [164, 165], have also been implemented to establish microwave radiation injury models, although nonmammalian studies are clearly less directly relevant to human effects.

## Methods for the assessment of microwave radiation injuries

### Methods for the evaluation of functional injuries

#### Brain function injuries

Numerous studies have reported that microwave radiation might influence brain functions [38, 65, 166]. Various methods were developed to investigate changes in brain function-related behavior, electrophysiological activities and BBB permeability. The methods used to study the harmful effects on brain functions induced by microwave radiation are described in the following sections.

1. **Behavior**

The behavioral methods used in studies on the biological effects induced by microwave radiation mainly focused on evaluating the function of learning and memory, anxiety, locomotor activity, depression, and excitability.

The Morris water maze (MWM), named after its inventor Richard Morris [167], was the most widely used behavioral method for learning and memory evaluation, especially for rodents [3, 4, 12, 13, 15, 16, 37, 40, 65, 68, 71, 73, 94, 110–112, 168–170]. In addition, other methods, such as the Y-maze, eight-arm radial maze, and elevated plus maze (EPM), were also used to study the effect induced by microwave exposure on learning and memory [2, 110, 112, 124, 171].

The most common methods to assess anxiety behavior induced by microwave exposure were the EPM and open field test (OFT) [71, 168, 172]. The EPM was designed based on the conflict between a rodent's instinct to explore a novel environment and their preference for closed arms. The EPM was widely used in anxiety assays [173]. As a popular behavioral test, the OFT is appropriate for social rodents with small living conditions. The OFT was used to measure anxiety-like behaviors in studies on microwave-radiation-induced injuries [71, 168, 174].

In addition, the behavioral tests commonly used to assess locomotor activities included the OFT [25, 124, 125], rotarod tests [125], and accelerated rotarod systems [124]. Moreover, forced swimming tests (FSTs) [124, 168] and tail suspension tests (TSTs) [168] were implemented to evaluate the level of depression induced by microwave radiation.

2) **Electrophysiological activities**

In studies on brain physiological activity changes caused by microwave radiation, the most commonly used methods were electroencephalography (EEG) and *in vivo* hippocampal long-term potentiation (LTP) recording. EEG could be used to reflect changes in brain function, including sleep quality [70, 166, 175–186]. LTP recording is a well-recognized electrophysiological method used to study synaptic plasticity induced by microwave radiation with respect to learning and memory [13].

3) **BBB permeability**

Evans blue (EB) staining was the most popular method used to study the changes in BBB permeability induced by microwave radiation.

Serum albumin is the main serum protein that cannot cross the BBB under physiological conditions. EB dye can bind to serum albumin tightly. Therefore, serum albumin can be traced by EB dye when using fluorescence microscopy. When BBB permeability increases, EB-dye-bound albumin may extravasate through the BBB into extracellular brain tissue [187]. It was reported that more EB dye was observed in brain tissue after microwave exposure [72, 90, 91, 188, 189].

In addition, several methods, such as albumin immunohistochemistry staining [89, 114–116, 190], transendothelial electrical resistance (TEER) measurement [38], horseradish peroxidase (HRP) staining method [38], and ^14^C-sucrose-tracing methods [148], could also be used to study the effects on BBB permeability induced by microwave radiation. TEER indicates the impedance to pass through the BBB, which is recognized as one of the most accurate and sensitive indicators of BBB integrity [38].

#### Reproductive function injuries

It was reported that microwave radiation might cause damage to reproductive functions, such as changes in sex hormone (such as testosterone and estradiol) levels and sperm parameters [7, 19, 191–193]. Enzyme immunoassays [122] and enzyme-linked immunosorbent assays (ELISAs) [19, 193–195] were widely used in studies of sex hormone levels after exposure to microwave radiation. A hemocytometer was implemented to assess spermatozoa motility and count [122, 191, 196]. Eosin-nigrosin staining and supra-vital staining could also be used to study sperm viability [122, 191, 192].

#### Cardiac function injuries

Many studies showed that microwave radiation might cause cardiac physiological (heart rate, blood pressure, etc.), biochemical (myocardial enzyme, ion concentration, etc.) and endocrine dysfunctions.

The most widely used method for evaluating the effect on cardiac physiological function following microwave radiation was electrocardiography (ECG) [197–199]. Furthermore, photoplethysmography (PPG) sensors and sphygmomanometers could also be used to investigate the effect of microwave radiation on heart rate and blood pressure [106, 200, 201]. Fluorescence was used to measure the changes in cardiac biochemical functioning induced by microwave radiation [79, 147]. Radioimmunoassay was the method of choice to evaluate cardiac endocrine function after exposure to microwave radiation [202].

#### Endocrine organ function injuries

There was evidence that microwave exposure might have negative impacts on endocrine organ function, mainly hormone level disorder. Many studies reported that ELISA was the most commonly used method for evaluating the effects of microwave radiation on endocrine organ function [2, 6, 193, 195, 203, 204].

### Methods for the evaluation of structural injuries

#### Microstructural injuries

With light microscopy, the morphological changes in the microstructure of either animal or cell models after exposure to microwave radiation could be observed using hematoxylin and eosin (HE) staining or special dyeing methods. HE staining is one of the most widespread methods for observing the microstructure of various organs, such as the brain, heart, reproductive organs, and endocrine organs [3, 4, 6, 12, 13, 15, 19, 67, 73, 79, 89, 92, 104, 107, 121, 205, 206]. The function of special dyeing methods is to demonstrate specific cellular components. Special dyeing methods were used to observe changes in specific cellular structures of nerve and testicular tissues in studies of microwave radiation effects [17, 40, 65, 92, 116, 207, 208].

Special dyeing methods used to analyze nerve tissue injury induced by microwave radiation included cresyl violet, toluidine blue, Fluoro-Jade B, Golgi, and Luxol fast blue staining. Cresyl violet and toluidine blue staining were designed to observe Nissl bodies in neurons [17, 116, 207]. De Gannes *et al*. [116] reported that both cresyl violet staining and Fluoro-Jade B methods indicated the occurrence of dark neurons and neuronal degeneration by observing the states of the Nissl bodies. This study suggested that the latter was a more reliable method of neuronal degeneration evaluation induced by microwave radiation. Golgi staining was used in dendritic spine density examination [40]. Luxol fast blue staining was implemented to observe nerve myelin [92].

The special dyeing method for examining the changes in testicular structure induced by microwave radiation was toluidine blue staining [208]. Toluidine blue staining was used for seminiferous tubule observation, which might be a simple method to observe spermatozoa [208].

#### Ultramicrostructural injuries

Electron microscopy, including transmission electron microscopy (TEM) [3, 4, 12, 13, 36, 65, 67, 79, 81, 104, 107, 209, 210] and scanning electron microscopy (SEM) [38], was used to observe ultrastructural changes in neurons, germ cells, and cardiomyocytes after microwave radiation.

### Methods for the investigation of the biological mechanisms of microwave exposure injuries

#### Apoptosis and abnormal proliferation

On the one hand, methods for mechanistic studies of microwave-radiation-induced apoptosis mainly included flow cytometry (FCM) [14, 68, 84, 155, 211, 212], in situ end labeling (TUNEL assay) [104, 107, 209, 213, 214], acridine orange/ethidium bromide staining (AO/EB) [14], and Muse cell analysis [79]. On the other hand, the widely used methods to analyze cell proliferation included MTT [155] and immunohistochemical assays [14, 25, 40, 191, 215, 216].

#### Cell membrane damage

The most commonly used method for the examination of ion channel activities after microwave radiation exposure was the whole-cell patch clamp technique [80]. Moreover, the fluorescence method was one of the most popular methods used to measure the changes in intracellular calcium ion (Ca^2+^) concentration induced by microwave exposure [67, 147, 160].

#### Changes in proteins

Various methods were used in mechanistic studies of microwave-radiation-induced changes in proteins, such as immunoassays, proteomic methods, and nondenaturing polyacrylamide gel electrophoresis (native PAGE).

1. **Immunoassays**
  Immunoassays based on antigen-antibody reactions are helpful in either qualitative or quantitative analysis of biological samples. The immunoassay methods implemented in the mechanistic studies of microwave radiation mainly included ELISA, Western blotting, immunohistochemistry, immunoprecipitation, and coimmunoprecipitation assays.
  ELISA is a classic method for measuring immunoreactions by binding soluble antigens or capturing antibodies on solid carriers [217]. ELISA was used to assess the changes in neurotransmitters, cytokines and protein kinase A (PKA) after microwave radiation exposure [74, 125, 203, 218].
  Western blotting is a widely used method in molecular biology, biochemistry, and immunogenetic studies. Western blotting could be used to measure the levels of protein expression, including stress-related proteins, apoptosis-related proteins, synapse-related proteins, signal transduction molecules, neurotransmitter receptors, etc. [37, 65, 73, 79, 219].
  Immunohistochemistry methods, such as radioimmunoassay, immunofluorescence, and immune colloidal gold techniques, can provide semiquantitative evaluation of proteins. They have the advantage of pinpointing the given antigen in the tissue. Immunohistochemistry technologies were used to study the biological effects of microwave radiation on neurotransmitter regulation, the stress response, cell proliferation and death regulation, cell membrane damage and signal transduction [2, 125, 191, 220].
  Diverse methods, including immunoprecipitation and coimmunoprecipitation, could also be used to evaluate the biological effects of microwave radiation on signal transduction molecules [38].
2. **Proteomics method**
  A proteome is an indication of a protein expression profile. Proteomics analyses compare protein expression levels and assess changes in protein patterns [221]. Proteomics analyses were used to examine the changes in proteins expressed in rat testes after exposure to 900 MHz microwave radiation, and two regulatory proteins, ATP synthase beta subunit and precursor, were found to be upregulated [222].
3. **Native PAGE**
  Native PAGE is both a qualitative measurement method and a protein separation and purification technique. Native PAGE maintains the activities of proteins without the addition of denaturants, such as sodium dodecyl benzene sulfonate or mercaptoethanol. Native PAGE combined with a spectrophotometric method was used to determine the activities of antioxidant enzymes after microwave radiation [41].

#### Changes in genes and gene expression

Gene evaluation methods used in biological mechanism studies of microwave radiation mainly include polymerase chain reaction (PCR), in situ hybridization (ISH), comet assays, electrophoretic mobility shift assays (EMSAs), DNA sequencing and genotyping methods.

PCR methods, such as real-time PCR and reverse transcription PCR (RT-PCR), have been widely used to investigate the effect of microwave radiation on the expression of genes involved in the stress response, signal transduction pathways, apoptosis, neurotransmitter receptors, cytokines, tight junction proteins, and restriction fragment length polymorphism (RFLP) analysis [14, 17, 18, 38, 68, 223, 224].

ISH was used to determine the expression of stress-related genes such as heat shock protein (HSP) 70 and c-fos mRNAs [225]. The comet assay is widely used to assess DNA damage [97, 191, 226]. EMSA was a technique used to investigate the interaction of DNA-binding proteins and their sequences. EMSA was used to examine the binding activities of a transcription factor and DNA after microwave exposure [68]. DNA sequencing was used to assess the variation in the promoter region of the 2B subunit of the N-methyl-D-aspartate receptor (NR2B) gene and analyze the relationships between brain damage and NR2B gene polymorphisms caused by microwave radiation [68].

Furthermore, both flow cytometry and confocal microscopy were used to assess the occurrence of micronuclei induced by microwave exposure [227].

#### Changes in oxidative stress parameters

The most popular methods for evaluating oxidative stress-related indicators after microwave radiation included colorimetry and electron spin resonance (ESR) technology.

Based on a reaction producing a colored substance, the colorimetric method is designed to analyze the contents of an unknown sample by measuring its color depth. In previous studies on the biological mechanism of microwave radiation, colorimetry was used to measure the level of malondialdehyde (MDA), total antioxidant status (TAS), total antioxidative capacity (TAC), and total oxidant status (TOS) [97, 216]. ESR is designed to determine the interactions between unpaired electrons and the environment. ESR was used to measure the rates of superoxide and nitrogen oxide generation caused by microwave radiation [23].

#### Changes in neurotransmitters

The most commonly used method in the measurement of neurotransmitters is high-performance liquid chromatography (HPLC). The development of HPLC is due to the introduction of the gas chromatography theory on the basis of classic liquid chromatography. In previous studies on the biological effects of microwave radiation, HPLC was widely used to measure the level of neurotransmitters such as aspartic acid (Asp), glutamate (Glu), glycine (Gly), and gamma-aminobutyric acid (GABA) [3, 12, 36, 67, 68, 73, 92, 124, 228].

## Indicators of microwave radiation-induced biological injuries

### Indicators of functional injuries

#### Indicators of brain function injuries

1. **Behavior**
  Behavioral indicators are frequently used to examine microwave radiation-induced abnormalities in brain functions, including learning and memory, anxiety, depression, and locomotor activity.
  Learning and memory, one of the most important cognitive functions, are hotspots in the field of microwave-radiation-induced biological effects. The most common behavioral indicators of learning and memory were swimming speed, average escape latency (AEL), the percentage of time spent in the target quadrant and average crossing times of the MWM, and the time to enter one of the closed arms of the EPM [3, 4, 12, 13, 15, 16, 37, 40, 65, 68, 71, 73, 94, 110–112, 168, 169]. Moreover, the behavioral indicators for the evaluation of anxiety and depression included the total distance traveled, the frequency of entries into and the duration of time spent in the center zone, the number of entries into all zones, the time spent in the periphery of the open field of the OFT, the percent frequency of entering the open arms, the percentage of time spent in the open arms of the EPM, and the immobility time on the TST and FST [71, 168]. The common behavioral indicators of locomotor activity mainly included the scores for the moving distance, moving duration and rearing frequency of the OFT [125].
  Previous studies showed that microwave radiation might negatively affect the learning and memory [2, 3, 65], anxiety, depression [168], and locomotor activity [125] of experimental animals. However, some studies reported that microwave radiation had no significant effect on learning and memory, anxiety, or depression [124].
2. **EEG**
  EEG can be used to depict the electrical activity of neurons in the brain. An encephalogram macroscopically indicates changes in brain function. In previous studies on the biological effects of microwave radiation, the indicators provided by an EEG mainly included spectral bands, gravity frequency, and power spectra [3, 4]. Hao *et al*. [3] reported that the power of α and δ waves of Wistar rats decreased and the power of θ waves increased after exposure to microwave radiation at an average power density of 30 mW/cm^2^ and a SAR value of 10.5 W/kg for 15 min per day, once every other day three times, which implied a perturbation in encephalogram activity.
3. **LTP**
  As a classic model designed for studies on learning and memory, LTP indicates the state of synaptic plasticity. The most popular indicator of LTP used in microwave radiation effect studies was the amplitude of population spikes (PSs) [13]. Wang *et al*. [13] reported a decrease in the amplitude of PSs in rats after microwave radiation exposure, which suggested defects in LTP induction and impairments in learning and memory.
4. **BBB permeability**
  Indicators used to depict BBB permeability in studies on microwave radiation biological effects included the presence of endogenous albumin in the brain [89, 114–116]; the expression of zonula occludens-1 (ZO-1) [38, 148], occludin [38], and glial fibrillary acidic protein (GFAP) [96, 148, 225]; TEER values [38], and the permeability coefficient of HRP [38]. The presence of endogenous albumin in the brain was the primary indicator used. ZO-1 and occludin are endothelial tight junction (TJ) proteins. The reduced expression of ZO-1 can disrupt TJ proteins and cause BBB breakdown. Tyrosine phosphorylation of occludin triggers BBB dysfunction [229]. GFAP, a marker of mature astrocytes, has been indicated to be responsible for maintaining astrocytic structure and shape [230]. The increased expression of GFAP indicates reactive astrocytes and brain injury [231]. A series of studies reported that microwave radiation might induce decreased expression of ZO-1 and occludin, enhanced tyrosine phosphorylation of occludin, and increased expression of GFAP in the brain [38, 96].
  In previous studies, it was found that BBB permeability increased after microwave radiation exposure [38, 114]. However, one study using head-only exposure of rats to the GSM-900 signal for 2 h showed no effect on BBB permeability [116].

#### Indicators of reproductive function injuries

The common indicators used in evaluations of reproductive function after microwave radiation included the level of testosterone, the level of estradiol, and sperm parameters (such as epididymal sperm motility, sperm concentration, vitality, sperm count, and the percentage of morphologically abnormal spermatozoa) [19, 191–193]. Recently, the available evidence was presented suggesting that microwave radiation exposure might have deleterious effects on the reproductive function of rats, e.g., decreased levels of serum testosterone; increased levels of estradiol; decreased sperm count, viability and motility; and increased sperm deformities [19, 191].

#### Indicators of cardiac function injuries

Previous studies showed that cardiac physiology, biochemistry and endocrine function can be adversely affected after microwave radiation.

The major indicators of cardiac physiological function implemented in studies of biological injuries induced by microwave radiation included heart rate, blood pressure and ECG [166, 198, 199, 232, 233]. It is well known that compensatory changes in blood pressure and heart rate occur in pathological processes. ECG indicators such as arrhythmia, heart block and myocardial infarction could be used in the diagnosis of heart diseases. Common blood pressure indicators used in studies on microwave biological effects included systolic blood pressure (SBP) and diastolic blood pressure (DBP) [232]. ECG indicators used in the studies on the biological effects of microwave radiation mainly included the time intervals between consecutive R waves and autonomic indices in both the time and the frequency domains, which depicted the measurement of the heart rate variability (HRV) [5, 166, 197, 198, 234–238].

Cardiac biochemical function indicators used in the studies of microwave-radiation-induced biological injuries mainly included myocardial enzyme spectrum levels and ion concentrations. It is well known that the activities of myocardial enzymes and intracellular or extracellular ion concentrations change when cardiomyocytes are injured and the integrity of the cell membrane is broken. The indicators of the myocardial enzyme spectrum used in previous studies of microwave-radiation-induced cardiac injuries mainly included the levels of lactate dehydrogenase (LDH), creatine kinase (CK), creatine kinase-MB (CK-MB) and hydroxybutyrate dehydrogenase (HBDH) [79, 205, 239]. The most commonly used indicator of ion concentration was the Ca^2+^ level of ventricular myocytes [80].

The heart can secrete various peptide hormones to regulate its own function. Therefore, the expression of these hormones could also be used to evaluate the state of cardiac endocrine function. The most popular indicator used in studies of microwave-radiation-induced cardiac injury is atrial natriuretic peptide (ANP) [8].

#### Indicators of endocrine organ function injuries

The indicators of endocrine organ function used in studies of injuries induced by microwave radiation mainly included the levels of plasma adrenocorticotropin hormone (ACTH), growth hormone (GH), cortisol (CS), corticosterone (CORT) and thyroid hormone (TH) [2, 6, 145, 172, 195, 204, 240]. ACTH and GH produced in the pars distalis of the adenohypophysis are involved in various pathophysiological processes, which are closely connected with the stress response. CS secreted by the zona fasciculata cells of the adrenal gland is a glucocorticoid with anti-inflammatory properties. The changes in TH induced by microwave radiation included changes in thyroxine (T4) and triiodothyronine (T3), which are synthesized by thyroid follicular epithelial cells and help to promote the development of the central nervous system (CNS) and metabolic function.

### Indicators of structural injuries

Indicators of brain structural injuries after microwave radiation mainly included cytological changes in neuronal components, such as the morphology of neurons, nuclei, cytoplasm (mitochondria, endoplasmic reticulum, etc.) and synapses. Increased numbers of degenerating neurons and stained nuclei and cytoplasm in the hippocampus were observed by light microscopy after exposure to microwave radiation [3, 12, 13, 15, 67, 71, 73, 121]. Changes in the cytoplasm in hippocampal neurons (mitochondria swelling and endoplasmic reticulum dilation) and synaptic structure (decreased density of synaptic vesicles, blurred synaptic gaps, and decreased postsynaptic density (PSD) length) were observed by electron microscopy [3, 65, 125].

The indicators used in the studies of structural damage of reproductive tissue induced by microwave radiation included the number of spermatogenic cells, the morphologies and diameter of seminiferous tubules, the thickness of the seminiferous epithelium and Leydig cells in testes, the diameter of the epididymis, and the height of the epithelium, the number of ovarian follicles [7, 19, 88, 191]. Azadi Oskouyi *et al*. [7] reported decreased epithelial height and diameter of the epididymis in New Zealand rabbits exposed to 950 MHz microwave radiation for 2 h/d for 2 weeks at an output power of 3 or 6 watts.

The indicators used in the studies on cardiac tissue structural effects induced by microwave radiation mainly included the morphology of cell nuclei and cytoplasm (mitochondria, glycogen granules and lipid droplets), the area fraction percentage of nonfibrotic myocardium, and the arrangement of myocardial fibers [8, 79, 107, 205]. Numerous histological results have indicated that microwave radiation might cause structural impairment in the heart, showing disordered muscle fibers, nuclear pyknosis, cytoplasmic vacuolization, myofilament impairment and reduction, decreased mitochondrion numbers, etc. [79, 107].

The indicators used in the studies of structural damage of endocrine tissue induced by microwave radiation included the thickness of the zona fasciculata (ZF), the cell size and perimeter of the ZF, and the columnar organization of ZF cells in the adrenal glands [6]. Shahabi *et al*. [6] reported that the fasciculata layer of the adrenal cortex thickened, the number of ZF cells was constant, and the ZF cell size and perimeter increased when Wistar rats were exposed to mobile radiofrequency (900 MHz) for 6 h/d for 4 ~ 8 weeks at a SAR of 1.010 W/kg.

### Indicators for investigations of the mechanism of biological injury

#### Neurotransmitters

The indicators involved in the studies of changes in neurotransmitters after microwave exposure mainly included amino acid neurotransmitters, choline neurotransmitters, catecholamine neurotransmitters, and their markers.

Amino acid neurotransmitters used in biological injury studies of microwave radiation mainly included inhibitory transmitters (GABA, Gly) and excitatory transmitters (Glu, Asp) [3, 12, 36, 37, 67, 68, 73, 124]. Choline neurotransmitters and their markers, such as acetylcholine (Ach), cholinesterase (ChE), and choline acetyl transferase (ChAT), were implemented in previous studies [239, 241]. Catecholamine neurotransmitters and their markers included dopamine (DA), noradrenaline (NA), serotonin (5-HT), tyrosine hydroxylase (TH), tryptophan hydroxylase (TPH), monoamine oxidase (MAO), and 3,4-dihydroxyphenylacetic acid (DOPAC), which were used in studies on the mechanism of brain injury induced by microwave radiation [18, 73, 92, 124, 125, 203, 204, 228]. However, there are still some controversies regarding the effects of microwave radiation on neurotransmitters. Some scientists argued that microwave radiation might irregularly alter the level of neurotransmitters in the brain [3, 68, 125, 241], whereas others did not find any changes [124].

#### Metabolic indicators

The metabolic indicators used in the studies of microwave-radiation-induced injuries mainly included ATP metabolism indicators (adenosine triphosphate (ATP), and CK) and mitochondrial function damage indicators (mitochondrial respiratory chain complexes I - IV, cytochrome oxidase (CO), etc.) [40, 79, 206, 242–244]. A series of studies demonstrated that energy metabolism disorders might be a cause of the adverse biological effect of microwave radiation, i.e., a significant decrease in the activities of CO, mitochondrial respiratory chain complexes I - IV and CK and the level of ATP [40, 79, 242].

#### Stress-related indicators

Stress-related indicators used in the studies of microwave radiation-induced injury mainly included all oxidative stress indicators, HSP70 levels, immediate early genes (such as c-fos and c-jun) and their protein levels, and endoplasmic reticulum stress indicators.

Oxidative stress, one of the most important mechanisms of microwave radiation-induced biological injuries, has been considered a result of the imbalance between pro-oxidant and antioxidant systems [19]. The common indicators implemented to examine oxidative stress included 1) free radicals (such as reactive oxygen species [ROS], nitric oxide [NO], and superoxide) [23, 40, 41, 156, 211, 226, 227, 245], 2) antioxidant indicators (enzymes such as superoxide dismutase [SOD], catalase [CAT], glutathione peroxidase [GSH-px], and non-enzymes [such as glutathione [246], TAC, TAS, and TOS]) [17, 19, 97, 191, 209, 247], and 3) oxidation products (such as MDA, conjugated dienes, protein carbonyl [PCO] and 8-hydroxydeoxyguanosine [8-OHdG]) [17, 45, 97, 191, 247]. A few reports indicated that free radicals might have adverse effects on cells and increase the oxidation of DNA bases, lipids, and proteins after microwave exposure [17, 45, 97, 191].

HSP70, a molecular chaperone, protects cells from various environmental stresses. The level of HSP70 was used to indicate the change in intracellular stress [110]. A series of studies demonstrated that microwave radiation with frequencies ranging from 900 MHz to 2450 MHz and power densities ranging from 50 mW/cm^2^ to 200 mW/cm^2^ could cause an increase in HSP70 levels in rat brain tissue, cardiomyocytes and chick embryos [110, 111, 206, 248].

The c-fos gene related to cell damage and even cell death can be induced to be expressed under nonpathogenic environmental conditions [225]. The morphological expression of c-fos is a biomarker of neuronal activation [220, 225]. Additionally, c-jun can be easily induced to be expressed under pathological conditions [225]. Several studies have reported increased expression of c-fos and decreased expression of c-jun in rat brains after exposure to microwave radiation [220, 225].

The indicators of endoplasmic reticulum stress used in studies of microwave radiation bioeffects mainly included the transcription factors XBP1, ATF4 and CHOP [224, 249]. A recent study showed that microwave radiation at frequencies between 900 MHz and 2450 MHz could decrease the expression of XBP1 splicing mRNA and increase the expression of ATF4 and CHOP mRNA in rat brains, suggesting the activation of endoplasmic reticulum stress [224].

#### Cell proliferation- and cell death-related indicators

The most popular indicators used in the studies of cell proliferation and cell death induced by microwave radiation were those involved in autophagy, apoptosis, inflammatory response and cell proliferation.

The commonly used autophagy indicators to evaluate the injuries of microwave radiation included microtubule-associated protein light chain 3 (LC3), the protein expression of autophagy-related gene (ATG) and lysosomal associated membrane protein 1 (LAMP1), and the ratio of LC3-II to LC3-I [3, 250, 251]. Although a series of studies were conducted focusing on autophagy and the biological effects of microwave radiation, the role of autophagy remains unclear.

The apoptosis-related indicators used in the studies of injuries of microwave radiation included Bcl-2 family proteins (such as the anti-apoptotic factor Bcl-2 and the pro-apoptotic factor Bax), apoptosis initiation factors (such as cytochrome C [Cyto C]), caspase family proteins (such as the apoptotic initiator caspase-9 and the apoptotic executor caspase-3), apoptosis rate, and pro-apoptotic gene p53 [14, 17, 40, 79, 191, 206, 215, 216, 227, 245].

The inflammatory-response-related indicators used in studies of injuries caused by microwave radiation included inflammatory cytokines, such as interleukin (IL)-1, IL-2, IL-6, IL-10, IL-12, tumor necrosis factor-α (TNF-α) and interferon-γ (IFN-γ) [45, 218, 223], and inflammatory genes, such as nuclear factor-kappa B (NF-κB) [191, 223]. The weight of evidence from studies on the inflammatory response supports the conclusion that inflammatory effects, including the increased expression of proinflammatory cytokines and the activated inflammatory pathway, might be a potential mechanism of injuries induced by microwave radiation [45, 218, 223].

The cell proliferation-related indicators commonly used in previous studies of microwave radiation injury effects included nucleoprotein Ki-67 and histone kinase [25, 211, 252]. Ki-67, as an endogenous marker of proliferation, was used to label proliferating cells [25]. The activity of histone kinase related to the G_2_/M phase transition in the cell cycle is increased in exponentially growing cells [211]. A few studies reported that the number of Ki-67-positive cells and the activity of histone kinase were decreased significantly after microwave exposure [25, 211, 252].

#### Indicators-related to cell membrane damage

The major indicators related to cell membrane damage that were implemented in the studies of injuries induced by microwave radiation included intracellular and extracellular ion concentrations, ion channel activity, and membrane receptor expression levels.

Changes in intracellular and extracellular ion concentrations can be used to depict the impairment of ion channels of the cell membrane and changes in cell membrane permeability. Intracellular Ca^2+^ is one of the most important ions for biological studies of microwave radiation and executes many biological processes [147]. A recent study reported that the levels of total calcium, endoplasmic reticulum calcium and mitochondrial calcium decreased after primary hippocampal neurons were exposed to 2.856 GHz pulsed microwave radiation, suggesting calcium efflux during microwave radiation exposure [147].

The activity of voltage-gated calcium channels (VGCCs) was used in previous studies as an indicator of microwave-radiation^-^induced changes in ion channels [80, 253]. Olgar *et al*. [80] found that although the L-type Ca^2+^ current (I_CaL_) values in cardiomyocytes were not altered after exposure to 2.1 GHz microwave radiation, the isoproterenol-induced I_CaL_ response was strikingly reduced.

The expression levels of N-methyl-D-aspartate receptor (NMDAR), β_1_-adrenergic receptor (β_1_-AR) and muscarinic type 2 acetylcholine receptor (M2-AChR) in the heart [12, 67, 254] have been used to evaluate the changes in cell membrane receptors induced by microwave radiation. It has shown that the expression levels of NMDARs at the postsynaptic membranes were related to excitatory synaptic transmission and synaptic plasticity [67]. The expression levels of β_1_-AR and M2-AChR were used to assess heart function [254].

Other membrane properties, such as the function of the synaptic vesicular membrane, can be indicated by the expression level of synaptic vesicular-associated proteins and the level of neurotransmitters [37, 69].

#### Signal transduction-related indicators

The signal-transduction-related indicators implemented in the studies on biological effects induced by microwave radiation mainly included 1) NMDAR-related signaling pathway molecules (such as the key NMDAR subunits, calmodulin-dependent protein kinase II [CaMKII], cyclic adenosine monophosphate [cAMP] responsive element-binding [CREB], postsynaptic density protein-95 [PSD-95], PKA and the p44/42 mitogen activated protein kinase [p44/42 MAPK]) [4, 12, 36, 67, 68, 74], 2) proteins of the protein kinase C (PKC) signaling pathway [84, 245, 255], 3) proteins of the signaling pathways associated with the activation of MAPK signaling cascades (such as extracellular signal-regulated kinases [ERK], c-jun amino-terminal kinases [JNK] and p38MAPK) [79, 227], and 4) proteins of the NO signaling pathway [80].

NMDAR-related signaling pathways are mainly involved in studies on the impairment of learning and memory function induced by microwave radiation [4, 12, 36, 67]. The activation of molecules in NMDAR-related signaling pathways has been suggested to be closely related to synaptic plasticity [12].

PKC is commonly used in biological studies of the effects of microwave radiation on the brain and male infertility [84, 245, 255]. PKC plays a critical role in cell signaling pathways to regulate cell proliferation, death, and stress. Studies have revealed that the decreased level of PKC in the brain and sperm cells might trigger an overproduction of ROS and subsequently cause injury after exposure [84, 245, 255].

It has been well established that MAPK cascades are responsible for regulating oxidative stress [79, 227]. Several studies found that microwave radiation might exert detrimental effects on the heart and brain through oxidative stress, which activates MAPK cascades [79, 227].

A study suggested that an upregulated NO signaling pathway might trigger a reduction in the β-adrenergic (β-AR) response of ventricular myocytes after microwave radiation exposure [80]. This was induced by modulating a second messenger, i.e., cyclic guanosine monophosphate (cGMP) [80].

#### Genotoxicity-related indicators

There are controversies regarding the genotoxic effects induced by microwave radiation exposure. Most studies have suggested that microwave radiation causes genotoxic effects [23, 97, 227, 256], while others have drawn different conclusions [257]. The genotoxicity-related indicators used in the studies of microwave radiation injury mainly included DNA single- or double-stranded breaks and micronuclei [23, 97, 227, 256].

## Discussion

In this review, we combined previous studies on microwave radiation biological effects to summarize the main factors that are essential for the establishment of a microwave radiation injury model: microwave exposure conditions, subjects used to establish injury models, and methods and indicators used to assess the establishment of injury models. The establishment of an injury model is the premise of studies on the biological effects of microwave radiation. The establishment of an injury model is important to both the reliability and the reproducibility of these studies, although the reproducibility may also be influenced by the funding sources of specific studies [54, 258]. Although fruitful results have been achieved, further research on the biological injuries induced by microwave radiation is an inevitable development trend.

First, 1) to establish a certain microwave radiation injury model (e.g., for the purpose of investigating the mechanisms of MW-induced bioeffects), a specified standardized microwave exposure procedure should be performed, which will be beneficial to comparative analyses of the results from different laboratories, although replication does not fail if the methodology is exactly the same as in previous studies. 2), to investigate the bioeffects induced by real-world microwave radiation, the exposure procedures should capture the complexity and diversity of real-world exposure conditions.

Second, although various kinds of subjects have been used to establish biological injury models of microwave radiation, hardly any uniform animal species and cell types are widely used in studies of the biological effects after microwave exposure. In addition, based on the sensitive biological indicators from previous studies, it is important to cultivate novel animal species and cell types sensitive to microwave radiation by transgenic technology.

Third, the choice of methods contributes to screening biological indicators with high sensitivity and specificity for microwave radiation-induced injuries. The methods used in studying the biological effects of microwave radiation rely on the development of science and technology. Appropriate techniques can facilitate studies on the biological effects caused by microwave radiation. In fact, to the best of our knowledge, most of the methods used in present studies cannot demonstrate the real-time biological changes induced by microwave radiation. In the future, the development of more *in vivo* methods will help us to screen more reliable and sensitive indicators.

Finally, there is a lack of recognized sensitive indicators of biological injuries caused by microwave radiation. Therefore, it is helpful to screen and verify the sensitive indicators provided by previous studies. This may create a new opportunity for diagnosis and therapeutic intervention. In the future, quantitative biomarkers should be further explored, which will lay a foundation for building a reliable dose-effect relationship of biological effects induced by microwave radiation.

## Conclusion

In summary, we reviewed the microwave exposure conditions, subjects used to establish injury models, and common methods and indicators used to establish injury models of microwave radiation. This work may be helpful for further studies of biological effects induced by microwave radiation.

## Data Availability

Not applicable.

## Abbreviations

5-HT: Serotonin
8-OHdG: 8-Hydroxydeoxyguanosine
Ach: Acetylcholine
Asp: Aspartic acid
ACTH: Adrenocorticotropin hormone
AEL: Average escape latency
ANP: Atrial natriuretic peptide
AO/EB: Acridine orange/ethidium bromide
ATG: Autophagy-related gene
ATP: Adenosine triphosphate
BBB: Blood-brain barrier
DBP: Diastolic blood pressure
BPS: Blood pressure
cAMP: Cyclic adenosine monophosphate
cGMP: Cyclic guanosine monophosphate
Ca^2+^: Calcium ion
CaMKII: Calmodulin-dependent protein kinase II
ChAT: Choline acetyl transferase
ChE: Cholinesterase
Cyto C: Cytochrome C
CAT: Catalase
CK: Creatine kinase
CK-MB: Creatine kinase-MB
CNS: Central nervous system
CO: Cytochrome oxidase
CORT: Corticosterone
CREB: Cyclic adenosine monophosphate responsive element-binding
CS: Cortisol
CW: Continuous wave
DA: Dopamine
DOPAC: 3,4-Dihydroxyphenylacetic acid
EB: Evans blue
ECG: Electrocardiography
ELISA: Enzyme-linked immunosorbent assay
EMSA: Electrophoretic mobility shift assay
EPM: Elevated plus maze
ERK: Extracellular signal-regulated kinases
ESR: Electron spin resonance
FCM: Flow cytometry
FST: Forced swimming test
Glu: Glutamate
Gly: Glycine
GABA: Gamma-aminobutyric acid
GFAP: Glial fibrillary acidic protein
GH: Growth hormone
GSH: Glutathione
GSH-px: Glutathione peroxidase
GSM: Global System for Mobile Communication
HBDH: Hydroxybutyrate dehydrogenase
HE staining: Hematoxylin and eosin staining
HPLC: High-performance liquid chromatography
HRP: Horseradish peroxidase
HRV: Heart rate variability
HSP: Heat shock proteins
ICaL: L-type Ca^2+^ currents
IFN-γ: Interferon-γ
IL: Interleukin
ISH: In situ hybridization
JNK: c-jun amino-terminal kinases
LAMP1: Lysosomal associated membrane protein 1
LC3: Microtubule-associated protein light chain 3
LDH: Lactate dehydrogenase
LTP: Long-term potentiation
M_2_-AChR: Muscarinic type 2 acetylcholine receptor
MAO: Monoamine oxidase
MDA: Malondialdehyde
MWM: Morris water maze
native PAGE: Nondenaturing polyacrylamide gel electrophoresis
NA: Noradrenaline
NF-κB: Nuclear factor-kappa B
NGF: Nerve growth factor
NMDAR: N-methyl-D-aspartate receptor
NO: Nitric oxide
NR2B: 2B subunit of N-methyl-D-aspartate receptor
OFT: Open field test
p44/42 MAPK: p44/42 mitogen activated protein kinase
PCO: Protein carbonyl
PCR: Polymerase chain reaction
PKA: Protein kinase A
PKC: Protein kinase C
PPG: Photoplethysmography
PS: Population spike
PSD: Postsynaptic density
PSD-95: Postsynaptic density protein-95
PW: Pulsed wave
RFLP: Restriction fragment length polymorphism
ROS: Reactive oxygen species
RT-PCR: Reverse transcription PCR
SAR: Specific absorption rate
SD rats: Sprague-Dawley rats
SEM: Scanning electron microscopy
SOD: Superoxide dismutase
SV40: Simian virus 40
T_3_: Triiodothyronine
T_4_: Thyroxine
TAC: Total antioxidative capacity
TAS: Total antioxidant status
TEER: Transendothelial electrical resistance
TEM: Transmission electron microscopy
TH: Thyroid hormone
TH: Tyrosine hydroxylase
TJ: Tight junction
TNF-α: Tumor necrosis factor-α
TOS: Total oxidant status
TPH: Tryptophan hydroxylase
TST: Tail suspension test
TUNEL: In situ end labeling
VGCCs: Voltage-gated calcium channels
WHO: World Health Organization
ZF: Zona fasciculata
ZO-1: Zonula occludens-1
β-AR response: β-adrenergic response
β_1_-AR: β_1_-adrenergic receptor
